# Effect of Inoculation with Arbuscular Mycorrhizal Fungi and Fungicide Application on the Secondary Metabolism of *Solanum tuberosum* Leaves

**DOI:** 10.3390/plants11030278

**Published:** 2022-01-21

**Authors:** Valentina Fritz, Gonzalo Tereucán, Christian Santander, Boris Contreras, Pablo Cornejo, Paulo Ademar Avelar Ferreira, Antonieta Ruiz

**Affiliations:** 1 Centro de Investigación en Micorrizas y Sustentabilidad Agroambiental, CIMYSA, Universidad de La Frontera, Avda. Francisco Salazar 01145, Temuco 4811230, Chile; v.fritz02@ufromail.cl (V.F.); g.tereucan01@ufromail.cl (G.T.); c.santander01@ufromail.cl (C.S.); pablo.cornejo@ufrontera.cl (P.C.); 2 Departamento de Ciencias Químicas y Recursos Naturales, Scientific and Technological Bioresource Nucleus BIOREN-UFRO, Universidad de La Frontera, Avda. Francisco Salazar 01145, Temuco 4811230, Chile; 3 Novaseed Ltda. and Papas Arcoiris Ltda., Loteo Pozo de Ripio s/n, Parque Ivian II, Puerto Varas 5550000, Chile; boriscontreras@novaseed.cl; 4 Departamento de Solos, Universidade Federal de Santa Maria, Santa Maria 97105-900, RS, Brazil; ferreira.aap@gmail.com

**Keywords:** potato, mycorrhiza, secondary metabolism, antioxidant activities, phenolic compounds

## Abstract

In potato (*Solanum tuberosum*) crops, the use of fungicides to control some diseases is widespread; however, it has been reported that this practice can modify the potato polyphenolic content, and new strategies oriented to the potato defense system are necessary. One alternative is the use of arbuscular mycorrhizal fungi (AMF) to improve the defense mechanisms of plants. In this study, phenolic profiles and antioxidant activities in leaves of three potato genotypes (CB2011-509, CB2011-104, and VR808) were evaluated in crops inoculated with three AMF strains (*Claroideoglomus claroideum, Claroideoglomus lamellosum,* and *Fumneliformis mosseae*) and with AMF in combination with the use of two commercial fungicides (MONCUT [M] and ReflectXtra [R]). Eight phenolic compounds were detected, mainly hydroxycinnamic acids (HCAD) and flavonols, in samples where the highest concentrations of HCAD were obtained, 5-caffeoylquinic acid was the most abundant phenolic. The antioxidant activity was higher using the cupric reducing antioxidant capacity (CUPRAC) and ferric reducing antioxidant power (FRAP) methods. The association of AMF with plants had benefits on the secondary metabolism; however, the response differed according to genotype. The different combinations of potato genotypes, AMF strain, and fungicide modified the content of phenolic compounds in leaves in different ways; the treatment using *C. lamellosum* and ReflectXtra was the ideal combination for the genotypes analyzed here, with the higher antioxidant response, which supports the further technological evaluation of efficient AMF strains and fungicides in potato crops.

## 1. Introduction

Potato (*Solanum tuberosum* L.) is one of the most widely cultivated plant species worldwide. According to the latest report from the Food and Agriculture Organization (FAO), total potato production was estimated at 381,682 thousand tons in 2014 [1] The quality of the tubers is negatively affected by substances such as glycoalkaloids, nitrates, and herbicide residues, which must not be present or must be present only in small quantities [2,3]. The nutritional value of this crop is associated with the chemical composition of the tuber, which includes starch, sugars, reducing sugars, proteins rich in essential amino acids such as leucine, lysine, phenylalanine, and threonine, dietary fiber, vitamins C, B1, B2, and B6, and several secondary metabolites with antioxidant capacity [2]. These antioxidants predominantly include polyphenols, flavonoids, polyamines, and carotenoids [4]. Several studies have shown that potato tubers are an important source of polyphenols [5,6,7]. The total phenolic content can also be modified by the environment and postharvest management [4]. However, the distribution of phenolic compounds varies throughout the plant. In the leaves, phenolic compounds are mainly represented by hydroxycinnamic acids and their conjugates (HCAD) with trans-5-O-caffeoylquinic acid being the most abundant [4,8]. Flavonols and flavones have also been reported in potato leaves, where 3’,4’-dihydroxyflavonol significantly inhibits the generation of superoxide radicals in a cell-free system and protects ascorbic acid from autooxidation [9]. However, despite the significant levels of phenolic compounds in leaves, its content of glycoalkaloids prevents its use as food due to its high toxicity with harmful effects on humans despite their protective activity against various pathogens and insects [10].

Recent research has focused on exploiting beneficial microorganisms in crop management, including arbuscular mycorrhizal fungi (AMF), which are widely distributed in soils and can colonize the roots of most plant species [11]. Arbuscular mycorrhizal fungi belong to the Glomeromycota phylum [12], which is widely recognized to improve plant growth through the increase of nutrient and water absorption by plant roots and also through the improvement of soil structure [11]. One of the most important benefits of AMF for its host plants is the protection against diseases from soil pathogens, including fungi, bacteria, and nematodes [13,14]. Arbuscular mycorrhizal fungi have been reported to effectively induce the accumulation of phenols and flavonoids, which are powerful antioxidants that act as free radical scavengers and reducing agents; in addition, AMF also increases gene expression related to secondary metabolism [15]. Arbuscular mycorrhizal fungi can also increase the expression of antioxidant compounds and enzymes and decrease the production of malondialdehyde (MDA), which is an indicator of damage to the plant membrane from lipid peroxidation [16].

In potato crops, the soil microorganism *Phytophthora infestans* is one of the most destructive diseases [17], in which the damage caused is extensive due to difficulties in controlling the spread of the disease. The use of chemical fungicides to reduce to *P. infestans* can provide some level of disease control [18]. Multiple fungicides can be used for this control. Among them, ReflectXtra is a preventive, systemic, and contact action fungicide that combines two different active ingredients (isopyrazam and azoxystrobin); both act at the level of mitochondrial respiration of fungi but at different parts of the process, representing a powerful resistance strategy. In contrast, *Rhizoctonia* spp. are another major fungal pathogen of potatoes, causing economic losses due to stem cankers and black scurf disease [19], MONCUT 40 SC is a systemic fungicide with preventive and curative action that efficiently controls *Rhizoctonia* spp., preventing their growth and penetration into the root and tuber structures, which causes collapse of the hyphae and stops their growth. Multiple fungicides can be used for this condition. However, it is important to consider that the use of fungicides can also negatively affect AMF populations in the same way [20,21,22]. Therefore, it is of great interest to study the response of plants to attack by pathogens or to abiotic changes at the metabolic and physiological levels. The use of fungicides is common and can benefit or harm the plant. Therefore, this study aimed to analyze the effect of fungicides on the symbiosis of AMF with potato plants and how this mycorrhizal-fungicide interaction can affect the defense system and the antioxidant response of the plant.

## 2. Results

### 2.1. Identification and Quantification of Phenolic Compounds

**Figure 1:**
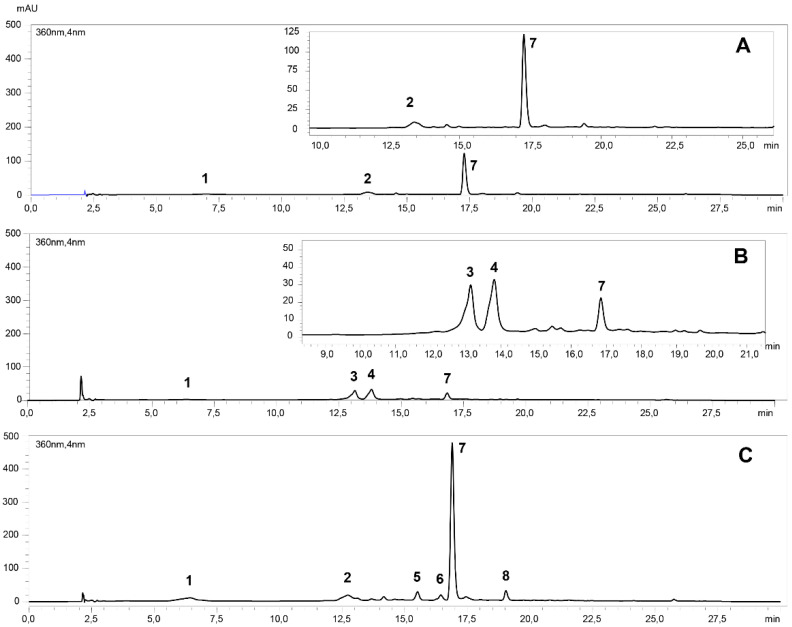
HPLC-DAD chromatogram at 360 nm of hydroxycinnamic acid derivatives and flavonols in *Solanum tuberosum* leaves under the treatment of fungicides and arbuscular mycorrhizal fungi, where: (**A**) VR808 genotype (**B**) CB2011-509 genotype (**C**), CB2011-104 genotype. For identification, please refer to details in Table 1.

Different profiles of phenolic compounds were detected among the genotypes. Specifically, in VR808, only three phenolic compounds were detected (Figure 1A, Table 1), which corresponded to two HCADs (5-caffeoylquinic acid (peak 1) and caffeoylquinic acid isomer (peak 2)) and the flavonol quercetin-rutinoside (peak 7). In CB2011-509, only one HCAD (5-caffeoylquinic acid) and three flavonols (3-glucosylrutinoside (peak 3), dihexoside (peak 4), and rutinoside (peak 7) derivatives of quercetin) were detected (Figure 1B, Table 1). Finally, for CB2011-104, a profile similar to VR808 was observed, but three other phenolic compounds were also detected (quercetin-pentoside-rutinoside (peak 5), kaempferol-rutinoside (peak 8), and another unidentified phenolic compound (peak 6)) (Figure 1C, Table 1).

**Figure 2:**
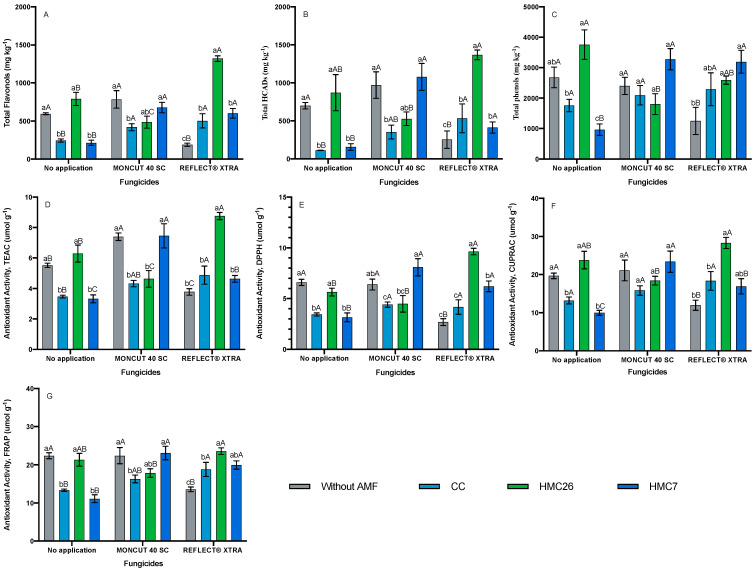
Phenolic compounds and antioxidant activity of *Solanum tuberosum* leaves, CB2011-104 genotype under inoculation of arbuscular mycorrhizal fungi and fungicide treatments. (**A**) Total flavonols by HPLC-DAD, (**B**) Total hydroxycinnamic acids by HPLC-DAD, (**C**) Total phenols determined by the Folin-Ciocalteu method, (**D**) AA determined by the TEAC (Trolox equivalent antioxidant capacity) method, (**E**) AA determined by the DPPH (2,2-diphenyl-1-picrylhydrazyl) method, (**F**) AA determined by the CUPRAC (copper reducing antioxidant capacity) method and (**G**) AA determined by the FRAP (ferric reducing antioxidant power) assay. Means followed by the same lowercase letter compare inoculation for the same fungicide and uppercase letters compare fungicides within the same inoculation condition (Tukey 5%). Where: without AMF, CC: *Claroideoglomus claroideum*; HMC26*: Claroideoglomus lamellosum* and HMC7: *Funneliformis mosseae*.

For the CB2011-104 genotype, higher concentrations of flavonols and HCADs were observed in the treatment inoculated with HMC26 combined with the use of the ReflectXtra fungicide (Figure 2A,B). In comparison, the treatments inoculated with HMC7 increased the phenolic concentrations under both MONCUT and ReflectXtra. Higher concentrations of 5-caffeoylquinic acid, quercetin-rutinoside, kaempferol-rutinoside, and quercetin-pentoside-rutinoside were observed when the plants were inoculated with HMC26 combined with the use of ReflectXtra fungicide (Figure S1).

**Figure 3:**
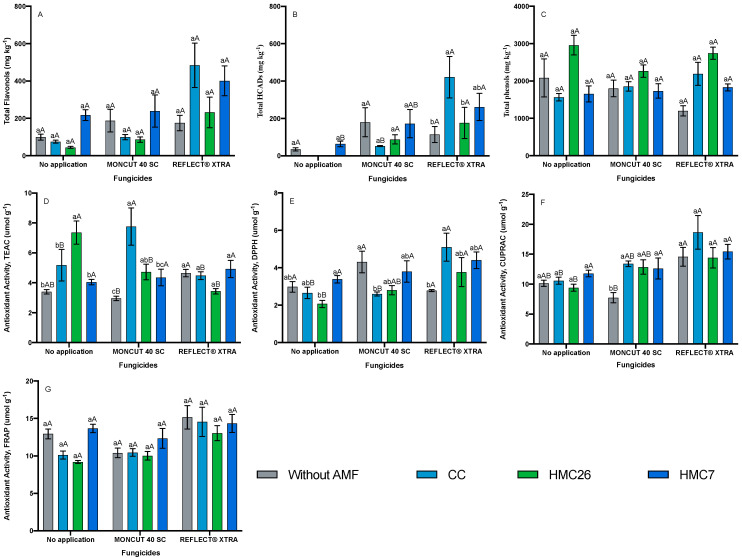
Phenolic compounds and antioxidant activity of *Solanum tuberosum* leaves, CB2011-509 genotype under inoculation of arbuscular mycorrhizal fungi and fungicide treatments. (**A**) Total flavonols by HPLC-DAD, (**B**) Total hydroxycinnamic acids by HPLC-DAD, (**C**) Total phenols determined by the Folin-Ciocalteu method, (**D**) AA determined by the TEAC (Trolox equivalent antioxidant capacity) method, (**E**) AA determined by the DPPH (2,2-diphenyl-1-picrylhydrazyl) method, (**F**) AA determined by the CUPRAC (copper reducing antioxidant capacity) method and (**G**) AA determined by the FRAP (ferric reducing antioxidant power) assay. Means followed by the same lowercase letter compare inoculation for the same fungicide and uppercase letters compare fungicides within the same inoculation condition (Tukey 5%). Where: without AMF, CC: *Claroideoglomus claroideum*; HMC26: *Claroideoglomus lamellosum* and HMC7*: Funneliformis mosseae*.

Contrary to the observations for CB2011-104, in the CB2011-509 genotype (Figure 3A,B), similar concentrations of HCADs and flavonols were observed in the treatment with CC and HMC7 combined with the use of the ReflectXtra fungicide (Figure 3A,B). However, there was a trend for increased total HCADs and flavanols in the presence of fungicides in the leaves of inoculated plants, mainly with the use of ReflecXtra. The use of MONCUT showed no significant increases in the concentrations of phenolic compounds in the leaves of AMF-inoculated plants. The CB2011-509 genotype showed the highest concentrations of 5-caffeoylquinic acid, quercetin-3-glucosylrutinoside, quercetin-dihexoside, and quercetin-rutinoside when inoculated with CC and HMC7 when the fungicide ReflecXtra was applied (Figure S2).

**Figure 4:**
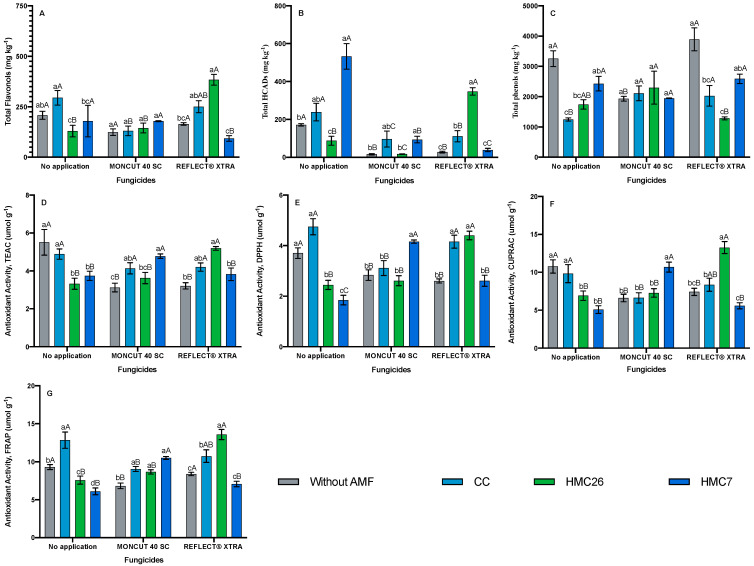
Phenolic compounds and antioxidant activity of *Solanum tuberosum* leaves, VR808 genotype under inoculation of arbuscular mycorrhizal fungi and fungicide treatments. (**A**) Total flavonols by HPLC-DAD, (**B**) Total hydroxycinnamic acids by HPLC-DAD, (**C**) Total phenols determined by the Folin-Ciocalteu method, (**D**) AA determined by the TEAC (Trolox equivalent antioxidant capacity) method, (**E**) AA determined by the DPPH (2,2-diphenyl-1-picrylhydrazyl) method, (**F**) AA determined by the CUPRAC (copper reducing antioxidant capacity) method and (**G**) AA determined by the FRAP (ferric reducing antioxidant power) assay. Means followed by the same lowercase letter compare inoculation for the same fungicide and uppercase letters compare fungicides within the same inoculation condition (Tukey 5%). Where: without AMF, CC: *Claroideoglomus claroideum*; HMC26: *Claroideoglomus lamellosum* and HMC7: *Funneliformis mosseae*.

In genotype VR808 (Figure 4A,B), similar concentrations of HCADs and flavonols were observed. The compound with the highest concentration was quercetin-rutinoside, reaching a concentration of 383.58 mg kg*^−^*^1^ when inoculated with HMC26 and with the use of ReflectXtra (Figure S3). In contrast, no increases were observed with the use of MONCUT compared with levels in the treatment without fungicide.

### 2.2. Total Phenols Concentrations

In the CB2011-104 genotype (Figure 2C), the highest concentration of total phenols was observed in the leaves of plants with HMC26 and without fungicide application, reaching 3756.8 mg kg*^−^*^1^. In the absence of AMFs, the phenol concentrations decreased when applying the fungicide ReflectXtra, whereas in the presence of AMFs, the total phenol concentrations tended to increase. Thus, the highest concentration of total phenols in leaves was in plants inoculated with HMC7 under the effect of fungicide, irrespective of whether the fungicide was MONCUT or ReflectXtra. In the CB2011-509 genotype (Figure 3C), a similar trend was observed; in the absence of AMF inoculation, the concentration of phenols decreased when fungicides were applied, whereas in the presence of AMF and treatments with fungicide, an increase in total phenol concentrations was observed. The VR808 genotype (Figure 4C) showed the highest concentrations of total phenols under the effect of ReflectXtra without AMF inoculation, reaching a total of 3891.7 mg kg*^−^*^1^. The highest concentration of total phenolics in the leaves of inoculated plants was under the treatment with HMC7 and with the use of ReflectXtra, reaching 2589.4 mg kg*^−^*^1^. Under inoculation with CC, the concentration of phenols increased under the effect of both fungicides. In contrast, inoculation with HMC26 increased phenolic compound concentrations only when MONCUT was used as a fungicide.

### 2.3. Antioxidant Activity

For the CB2011-104 genotype, there was a decrease in antioxidant activity in the treatments without mycorrhiza when applying the fungicide ReflectXtra. In the leaves of plants treated with HMC26 under the effect of ReflectXtra compared with the treatments without fungicide, an increase in antioxidant activity of 18 to 71% was observed (Figure 2D–F). The CUPRAC and FRAP activities exhibited lower increases relative to the other activities; however, both methods exhibited higher antioxidant activities.

In the CB2011-509 genotype, the antioxidant activity determined using TEAC was higher with CC inoculation combined with MONCUT fungicide application (Figure 3D). The activity of DPPH and CUPRAC was higher in plants that were inoculated with CC and received the application of the fungicide ReflectXtra. For CUPRAC antioxidant activity (Figure 3F), an increasing trend was observed in all treatments, where higher values were observed in the leaves of plants inoculated with CC under the use of ReflectXtra, with a 75% increase over the treatment without fungicide. The FRAP antioxidant activity for this genotype (Figure 3G) was also high for all treatments, particularly for the leaves of plants inoculated with HMC7 and CC under the use of ReflectXtra. The VR808 genotype (Figure 4D–G) showed a similar trend to the CB2011-104 genotype. In general, increases in antioxidant activity were observed in the leaves of plants inoculated with AMF, particularly when fungicides were applied. For the activities of CUPRAC (Figure 4F) and FRAP (Figure 4G), there were increases of 92% and 78% compared with those of the control, respectively, when the fungicide ReflectXtra was applied in the presence of HMC26.

### 2.4. Multivariate Analysis

**Figure 5:**
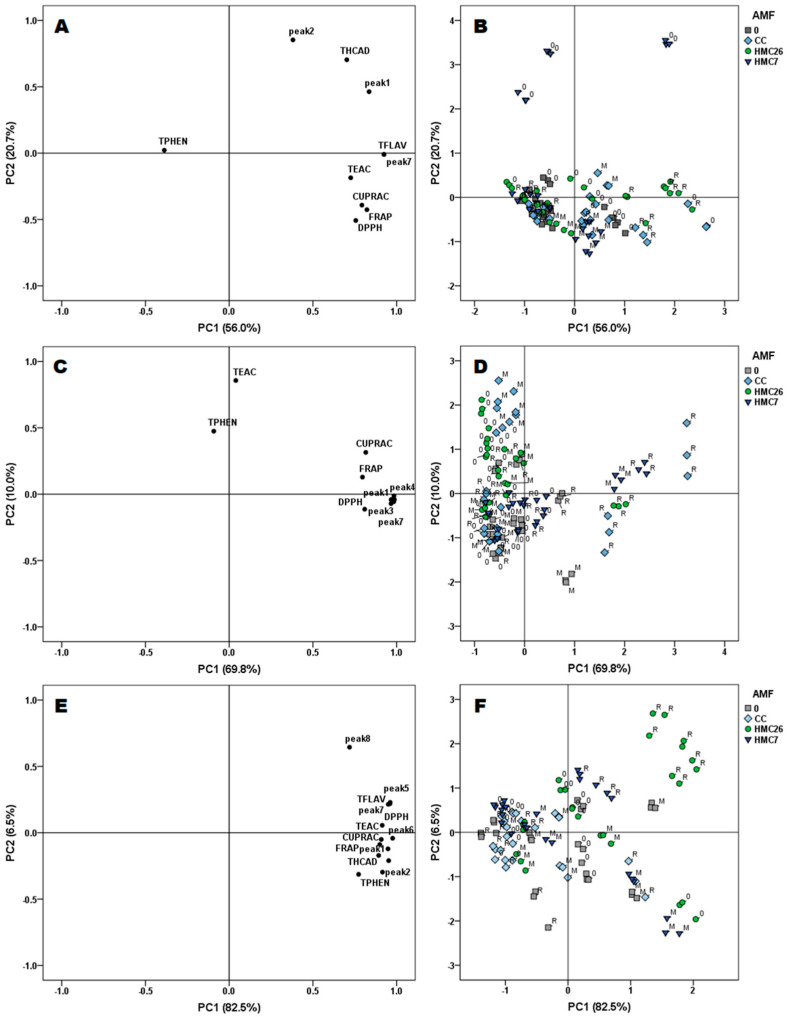
Scores of the principal component (PC) for the experimental variables (**left**) determined in leaves of three genotypes of *Solanum tuberosum* and the grouping of the samples according to the PCs distribution (**right**). VR808 (**A**,**B**), CB2011-509 (**C**,**D**), CB2011-104 (**E**,**F**). Where: peak 1: 5-caffeoylquinic acid; peak 2: caffeoylquinic acid; peak 3: quercetin-3-glucosylrutinoside; peak 4: quercetin-dihexoside; peak 5: quercetin-pentoside-rutinoside; peak 6: no identified; peak 7: quercetin-rutinoside; peak 8: kaempferol-rutinoside; TFLAV: total flavanols; THCAD: total hydroxycinnamic acids; TPHEN: concentration of total phenolics according to Folin–Ciocalteu method; TEAC: Trolox equivalent antioxidant capacity; CUPRAC: reducing antioxidant capacity of the cupric ion; DPPH: antioxidant activity of the DPPH radical; FRAP:, ferric reducing antioxidant power; 0: without AMF; CC: *Claroideoglomus claroideum*; HMC26: *Claroideoglomus lamellosum*; HMC7: *Funneliformis mosseae*; M: MONCUT and R: ReflectXtra.

A principal component (PC) analysis was performed separately for each potato genotype (Figure 5). In the VR808 genotype (Figure 5A), the most representative variables for PC1 were flavonols, HCADs, and antioxidant activities, in which quercetin-rutinoside was the main compound within the total flavonols. Additionally, a high correlation between TEAC, CUPRAC, FRAP, and DPPH was observed. The above dataset was mainly associated with the groups including HMC26 and CC with the use of ReflectXtra fungicide (Figure 5B). This could indicate that the AMF-plant interaction is beneficial for the antioxidant response in this genotype, even when a specific fungicide, ReflectXtra, is used. It was also observed that the plants inoculated with CC in the presence of the fungicide ReflectXtra induced the best antioxidant response, higher yield, and a greater mycorrhizal presence (data not shown).

For the CB2011-509 genotype, the most representative variables of PC1 were 5-caffeoylquinic acid, quercetin-3-glucosylrutinoside, quercetin-dihexoside, and quercetin-rutinoside, which were strongly correlated with the antioxidant activities of DPPH, CUPRAC, and FRAP (Figure 5C). For the above variables, the treatments with HMC26 and CC were generally grouped. Moreover, ReflectXtra showed a high association with plants colonized by HMC26 (Figure 5D). Finally, the CB2011-104 genotype showed similarities between the most representative variables of PC1, because flavonols and HCADs were strongly correlated (Figure 5E). In addition, there was a high correlation of these compounds with the total phenolic content, and CUPRAC, TEAC, FRAP, and DPPH antioxidant activities. Regarding the grouping of experimental individuals, the most representative treatments were the mycorrhizae HMC26 and CC combined with the use of the fungicide ReflectXtra (Figure 5F).

## 3. Discussion

Arbuscular mycorrhizal symbiosis activates the defense mechanism in host plants, increasing the antioxidant activity of phenolic compounds and improving plant growth [23,24]. Fungicide application may influence AM symbiosis, although the results are quite varied depending on the active ingredient, dose, timing of application, mode of application, and environmental conditions [19,20]. Additionally, it is well documented that plant genotype can influence AM symbiosis [19,21], similar to the use of commercial fungicides as MONCUT or ReflectXtra [22]. According to Hage-Ahmed et al., (2019) [21], some AMF species are more resistant to certain fungicides, whereas others appear to have a neutral effect on AM colonization, and others seem to influence AM symbiosis in a positive manner. 

Potato plants inoculated with AMF, mainly *C. claroideum*, showed a tendency to have increased phenolic compound concentrations under both MONCUT and ReflectXtra. In other similar studies, higher antioxidant activities and phenol contents were detected in AM-colonized lettuce, artichoke, and tomato plants [23,25,26]. It is well known that AMF stimulates phenolic compound synthesis in host plants, which becomes more important when plants are subjected to abiotic or biotic stress [27]. In contrast, Santander et al., (2020) [22] reported a higher concentration of phenolic compounds in non-inoculated lettuce plants, which was associated with high oxidative stress levels under saline conditions. Similarly, no significant differences in total polyphenol concentration were reported in the fruits of *Fragaria ananassa* plants inoculated or non-inoculated with the fungus *C. claroideum* [24]. However, differences in polyphenol content depending on the AMF strain have been reported, as not all varieties appear to be functionally compatible with the same fungus [28]. Most species of AMF may associate with many plant species, and a single plant may be colonized by several different species of AMF. Nevertheless, the responses of plants and their AMF may be different, suggesting different degrees of functional compatibility between specific AMF strains and plant species [29]. Notably, phenolic acids and flavonoids are involved in plant defense mechanisms, increasing plant resistance to fungal attack and inhibiting hyphal development [30]. In this context, in our study, phenolic compound concentrations were also increased, mainly by AM symbiosis.

The active compound in ReflectXtra is azoxystrobin, which is an analogue compound of strobilurin [31]. It has been shown that the strobilurin group of fungicides, in addition to their antifungal activities, can modulate plant physiology and biochemistry [32]. According to Schiavon et al., (2021) [33], the positive effects on plants include an increase in chlorophyll content, photosynthetic activity, stomatal aperture, water consumption, plant antioxidant activity, etc. In blackcurrant plants under applications of pyraclostrobin and strobilurin, an increase in phenolic compound concentrations has been reported [34]. In our study, the use of ReflectXtra showed significant increases in total flavonols and total HCADs in leaves, mainly in the VR808 and CB2011-509 genotypes.

The protection of the plant by AMF is known as mycorrhizal-induced resistance (MIR). This resistance has been associated with metabolic and genetic rearrangement that occurs due to the colonization of roots by AMF, which affects the plant’s primary and secondary metabolism [35]. The alteration of plant metabolism caused by AMF can increase the synthesis of some defense enzymes and is the most important mechanism of physiological and biochemical plant resistance. It has been shown that several plant metabolic pathways are altered after AM root colonization, particularly phenol alcohols and oxylipin derivatives [35]. Apparently, plant-AMF symbiosis affects root and leaf gene expression [36], causing metabolic changes in plants [37]. This result is consistent with that described in lettuce plants, where AM symbiosis with *C. lamellosum* and *F. mosseae* was demonstrated, improving nutrient uptake and photosynthetic capacity and increasing the defense mechanism in plant leaves [21,38,39]. Chen et al. (2013) [15] suggested that AMF effectively induces the accumulation of phenols and flavonoids because these are powerful antioxidants that act as free radical scavengers and reducing agents. In this study, for all potato genotypes, the methods with the highest antioxidant activities were CUPRAC and FRAP. These activities have great antioxidant power, which could be increased by the association with AMF for the different genotypes [25]. In a similar study where the antioxidant activity was evaluated by the FRAP method in the leaves of artichoke plants inoculated with *C. claroideum*, it was observed that the antioxidant activity was increased [40]. Moreover, Ruiz et al. (2019) [41] observed a higher antioxidant activity in *F. ananassa* fruits using the TEAC method compared with our results, but higher levels of anthocyanins were detected. This result could be explained because this method better reflects the antioxidant activity based on anthocyanins than on flavonols and HCADs [41]. Our results agree with other studies in which the activities of TEAC, DPPH, and CUPRAC were determined in *F. ananassa* inoculated with *C. claroideum*, where an increase in these antioxidant activities was observed [24]. However, Santander et al. (2020) [22] showed a decrease in antioxidant activities in lettuce plants inoculated with *C. claroideum*, and associated a higher accumulation of phenolic compounds and antioxidant activities with yellowing and browning of leaves as a result of leaf senescence in non-inoculated plants.

Our data clearly shows an increase in the secondary metabolites in potato leaves under the effect of fungicides, particularly under inoculation with HMC26 and the fungicide ReflectXtra, which was the treatment that showed the strongest effects on phenolic compound concentrations and antioxidant activities. This increase in the synthesis of phenolic compounds and antioxidant activity due to AMF and fungicide interactions may provide greater tolerance of *Solanum tuberosum* plants to infection by pathogenic fungi such as *Phytophthora infestans* and *Rhizoctonia* spp. The active components of ReflectXtra (isopyrazam and azoxystrobin) are characterized by affecting electron transference at the mitochondrial level, influencing metabolism and reducing oxidative stress [42]; the combination of both components could enhance these characteristics, ultimately showing greater effectiveness than the use of MONCUT, which contains only flutolanil. The fungicide ReflectXtra showed a positive effect on the leaves of *Solanum tuberosum* plants inoculated with AMF in most of the treatments. However, it would be interesting to evaluate other fungicides with similar characteristics on this plant and to analyze other mechanisms that could benefit from inoculation with AMF under the effect of fungicides in plants and also in tubers [19].

## 4. Materials and Methods

### 4.1. Samples

*Solanum tuberosum* plants were cultivated in pots in an open area subjected to ambient conditions but under artificial shadow using a 50% mesh outside the greenhouse of the Departamento de Ciencias Químicas y Recursos Naturales, Universidad de la Frontera, Temuco, Chile. Three genotypes (VR808, CB2011-509, and CB2011-104) with different skin and pulp colors were provided by Papas Arcoiris Ltd.a. (Puerto Varas, Chile). Each genotype was examined in a separate experiment. For the study, a completely randomized 4 × 3 factorial design with three replicates was performed. The treatments consisted of inoculation with three AMF (CC: *Claroideoglomus claroideum*; HMC26: *Claroideoglomus lamellosum*, and HMC7: *Funneliformis mosseae*, and one control without AMF inoculation) and the application of two commercial fungicides (MONCUT 40 SC [M] and REFLECT**^®^** XTRA [R] and one without fungicide application). *F. mosseae* and *C. lamellosum* were isolated from the rhizosphere of *Baccharis scandens* (Ruiz and Pav.) (Asteraceae) plants in the Camiña Valley, Atacama Desert (Tarapacá Region, northern Chile), and the fungus *C. claroideum* was isolated from agricultural soils from the Araucanía Region (Chile) and was associated with the rhizosphere of wheat plants. All of them were identified through PCR analysis performed for the partial small subunit (SSU) ribosomal RNA using the primers NS31 and AML2 [29]. In all cases, the reproduction of all AM fungal species was performed in trap pots using *Bidens pilosa* as host plant for six months, this substrate was used as AMF inoculum. The AM fungal inoculum was placed in the growth substrate under the tuber in amounts of 5 g per pot (approximately 700 spores per gram). The non-mycorrhizal microbiota was reconstituted by the application of 10 mL of a filtrate that contained 50 g each of inoculum and soil in 2000 mL sterile distilled water that was filtered through Whatman No 1 filter paper. The sowing was conducted on 12 September 2019. Sterilized peat, which was autoclaved for 20 min at 121 °C and 1 atm of pressure, was used as the substrate for the 108 11 L pots. All potato leaf genotypes were harvested on 14–15 January 2020 and were immediately stored at −80 °C until analysis.

### 4.2. Identification and Quantification of Phenolic Compounds

The identification of phenolics was carried out as described by Aguilera et al. 2020 [43], with minor modifications. First, the leaf samples were immersed in liquid nitrogen, 0.3 g were mashed, and 5 mL of extraction solvent (methanol:formic acid 95:5 *v*:*v*) was added in darkness. The samples were subsequently sonicated with an ultrasonic processor at 130 W (Sonics and Materials, Connecticut, USA) for 60 s at 40% amplitude, shaken for 30 min at 200× *g* and finally centrifuged (Lab Companion, Korea) for 10 min at 4000× *g*. Finally, the supernatant was transferred to another tube, protected of the light, and stored at −20 °C until the antioxidant measurements were made. For the HPLC determinations, the extract was filtered with 0.45 µm pore filters and injected into amber vials.

The HPLC analyses were performed according to the method described by Santander et al. (2020) [22] using high-performance liquid chromatography with diode array detection (HPLC-DAD) equipped with a quaternary pump (LC-20AT), a DGU-20A5R degassed unit, a CTO-20A oven, an SIL-20a autosampler and a UV–visible diode array detector (SPD-M20A) (Shimadzu, Tokyo, Japan). Data collection was carried out using Lab Solutions software (Shimadzu, Duisburg, Germany). Identity assignments were performed using an HPLC-DAD system coupled to an Applied Biosystem MDS Sciex system QTrap3200 LC/MS/MS mass spectrometer (Foster City, CA, USA). The data were collected using Analyst software (v.1.5.2) (SCIEX, Woodlands Central Indus. Estate, Singapore) for MS/MS analysis. The chromatographic separation method for the determination of phenolics, mainly hydroxycinnamic acids and flavonols, was performed based on the methods of Santander et al. (2020) [22], with minor modifications, using a Kromasil Classic-Shell C_18_ column (100 × 4.6 mm, 2.5 µm) and a Novapak precolumn Waters C_18_ (22 × 3.9, 4 µm). The samples were injected at a temperature of 40 °C and a flow rate of 0.55 mL min**^−^**^1^. The mobile phases used were 92:3:5 (*v*:*v*:*v*) water:acetonitrile:formic acid (mobile phase A) and 45:50:5 (*v*:*v*:*v*) water:acetonitrile:formic acid (mobile phase B) with an elution gradient from 6 to 30% B in 10 min, 30 to 50% B in 9 min, and 50 to 6% B in 2 min, followed by 10 min of stabilization. The detection wavelengths were 320 nm for HCADs and 360 nm for flavonols using chlorogenic acid and quercetin as standards for external calibration, respectively.

### 4.3. Determination of Total Phenols Using the Folin-Ciocalteu Method

The reagents were added in the following order to a microtube: 15 µL of standard or extract, 750 µL of deionized water, 75 µL of Folin-Ciocalteu reagent, 300 µL of sodium carbonate 20% m/v, and 360 μL of deionized water. The solutions were incubated at 20 °C for 30 min in the dark; 250 μL of the solution was then added to a 96-well plate, and the absorbance was read at 750 nm [24]. The tests were performed using chlorogenic acid as a standard, in which the concentrations of the calibration curve corresponded to 100, 200, 300, 400, and 500 mg L**^−^**^1^.

### 4.4. Antioxidant Activity Determinations

Trolox equivalent antioxidant activity (TEAC) is based on the discoloration of the radical cation ABTS^+^ in the presence of electron donor molecules. The chromophore is produced by the oxidation of ammonium 2,2′-azino-bis-(3-ethyl)-benzothiazoline-6-sulfonate (ABTS) with potassium persulfate, which produces an intense blue color. Before use, the solution must be adjusted to an absorbance of 0.7 (734 nm) by dilution with ethanol or water [44]. A total of 245 µL of ABTS^+^ 7.5 mM reagent (absorbance 1) and 5 µL of sample or Trolox standard were mixed in microplate wells. The samples were then incubated at 30 °C for 30 min (absorbance 2). The measurements were subsequently conducted at a wavelength of 734 nm, and the results were expressed as Trolox equivalents [24]. 

For cupric ion reducing antioxidant activity (CUPRAC), the following mix: 50 µL of 10 mM CuCl_2_, 50 µL of 7.5 mM neocuproine and 50 µL of 1 M ammonium acetate buffer at pH 7, were added to a 96-well plate and incubated at 27 °C for 15 min. Next, 100 μL of Trolox standard or sample was added and incubated for 30 min at 27 °C. The determinations were made at 450 nm, and the results were expressed as Trolox equivalents [45]. 

The determination of antioxidant activity using DPPH was based on the methodology described by Maldonado et al., (2005) [46], with some modifications. Briefly, 240 µL of 0.1 mM DPPH radical dissolved in methanol was added to a 96-well plate, where the first absorbance was read. Next, 10 µL of sample or standard was added and incubated for 30 min in the dark, and the second absorbance was read. Measurements were conducted at 517 nm, and the results were expressed as Trolox equivalents. 

The determination of antioxidant capacity using ferric reducing antioxidant power (FRAP) assay was conducted as described by Jiménez-Aspee et al. (2014) [47], with some modifications. Briefly, the FRAP solution was prepared by mixing 300 mM acetate buffer, 10 mM TPTZ, and 20 mM FeCl_3_ at a 10:1:1 *v*:*v*:*v* ratio. Next, 285 µL of FRAP solution heated to 37 °C was transferred to a 96-well plate followed by 15 µL of sample or Trolox standard with an incubation time of 30 min at the same temperature and in the dark. The absorbance was measured at 593 nm. The results were expressed as Trolox equivalents.

### 4.5. Statistical Analysis

Prior to the analysis of variance (ANOVA) and when necessary to meet the assumptions of normality and homoscedasticity, all data were transformed using the ln function, but the results are presented in their original scale of measurement. Treatments with significant differences were analyzed using Tukey HSD as a post hoc test to compare the means between treatments. The data were also subjected to a principal component analysis (PCA) to evaluate the multivariate effect of the established treatments and the relationship between experimental variables. For all the procedures, we considered *p* < 0.05 to be statistically significant. The software SPSS 22.0 (IBM Corp.) was used for the analyses. 

## 5. Conclusions

This work aimed to analyze the inoculation of arbuscular mycorrhizal fungi (AMF) on three genotypes of *Solanum tuberosum* based on physiological and metabolic responses and to establish the effects that common fungicides used on this crop have on AMF. Our results showed clear beneficial effects on secondary metabolism in plant leaves, which were mainly dependent on the AMF strain and the fungicide used. This result is of technological interest because the metabolic response is represented by antioxidant activities and profiles of secondary metabolites, and antioxidant potential has emerged as a tool to describe the compatibility among specific AMF strains and fungicides. Moreover, the use of different fungicides showed clear differences, with ReflectXtra being the alternative that noticeably increased secondary metabolism, mainly in the AMF-inoculated treatments. The different results obtained were related to the different combinations of fungi, potato genotype, and fungicide, which affected the content of antioxidant compounds in the plant; under our conditions, the interaction among *C. lamellosum* and the fungicide ReflecXtra, in generating a desirable secondary metabolite profile, was the best treatment for all potato genotypes.

## Figures and Tables

**Table 1:** Identifications of phenolic compounds in *Solanum tuberosum* leaves by HPLC-DAD-ESI-MS/MS.

Peak	t_R_ (min)	Identifications	Λmax (nm)	[M-H]^-^	Product Ions
1	6.4	5-caffeoylquinic acid	324	353.0	190.9
2	13.0	caffeoylquinic acid	327	353.9	191.0; 179.9; 173.0; 135.0
3	13.3	quercetin-3-glucosylrutinoside	352	771.8	300.1
4	13.9	quercetin-dihexoside	352	624.9	300.1
5	15.1	quercetin-pentoside-rutinoside	351	740.9	300.1
6	16.4	no identified	351		
7	16.9	quercetin-rutinoside	353	609.0	300.1
8	19.6	kaempferol-rutinoside	345	592.9	284.9

## Data Availability

The data presented in this study are available on request from the corresponding author.

## Supplementary Materials

The following supporting information can be downloaded at: https://www.mdpi.com/article/10.3390/plants11030278/s1, Figure S1: Phenolic compounds concentrations by HPLC-DAD in *Solanum tuberosum* leaves, genotype CB2011-104 under inoculation of arbuscular mycorrhizal fungi and fungicide treatments. Means followed by the same lowercase letter compare inoculation for the same fungicide and uppercase letters compare fungicides within the same inoculation condition (Tukey 5%). (A) 5-caffeoylquinic acid (peak 1), (B) caffeoylquinic acid isomer (peak 2), (C) quercetin-pentoside-rutinoside (peak 5), (D) no identified (peak 6), (E) quercetin-rutinoside (peak 7), (F) kaempferol-rutinoside (peak 8). Figure S2: Phenolic compounds concentrations by HPLC-DAD in *Solanum tuberosum* leaves, genotype CB2011-509 under inoculation of arbuscular mycorrhizal fungi and fungicide treatments. Means followed by the same lowercase letter compare inoculation for the same fungicide and uppercase letters compare fungicides within the same inoculation condition (Tukey 5%). (A) 5-caffeoylquinic acid (peak 1), (B) quercetin-3 glucosylrutinoside (peak 3), (C) quercetin-dihexoside (peak 4), (D) quercetin-rutinoside (peak 7). Figure S3: Phenolic compounds by HPLC-DAD in *Solanum tuberosum* leaves, genotype VR808 under inoculation of arbuscular mycorrhizal fungi and fungicide treatments. Means followed by the same lowercase letter compare inoculation for the same fungicide and uppercase letters compare fungicides within the same inoculation condition (Tukey 5%). (A) 5-caffeoylquinic acid (peak 1), (B) caffeoylquinic isomer (peak 2), (C) quercetin-rutinoside (peak 7).
