# Supplementary material for: Effect of Inoculation with Arbuscular Mycorrhizal Fungi and Fungicide Application on the Secondary Metabolism of Solanum tuberosum Leaves
Source: Plants (Basel). 2022 Jan 21;11(3):278. doi: 10.3390/plants11030278 (PMC8838535; doi:10.3390/plants11030278)
Supplement: Supplementary file 1 [file plants-11-00278-s001.zip › plants-1543325-Suplemmentary.pdf]

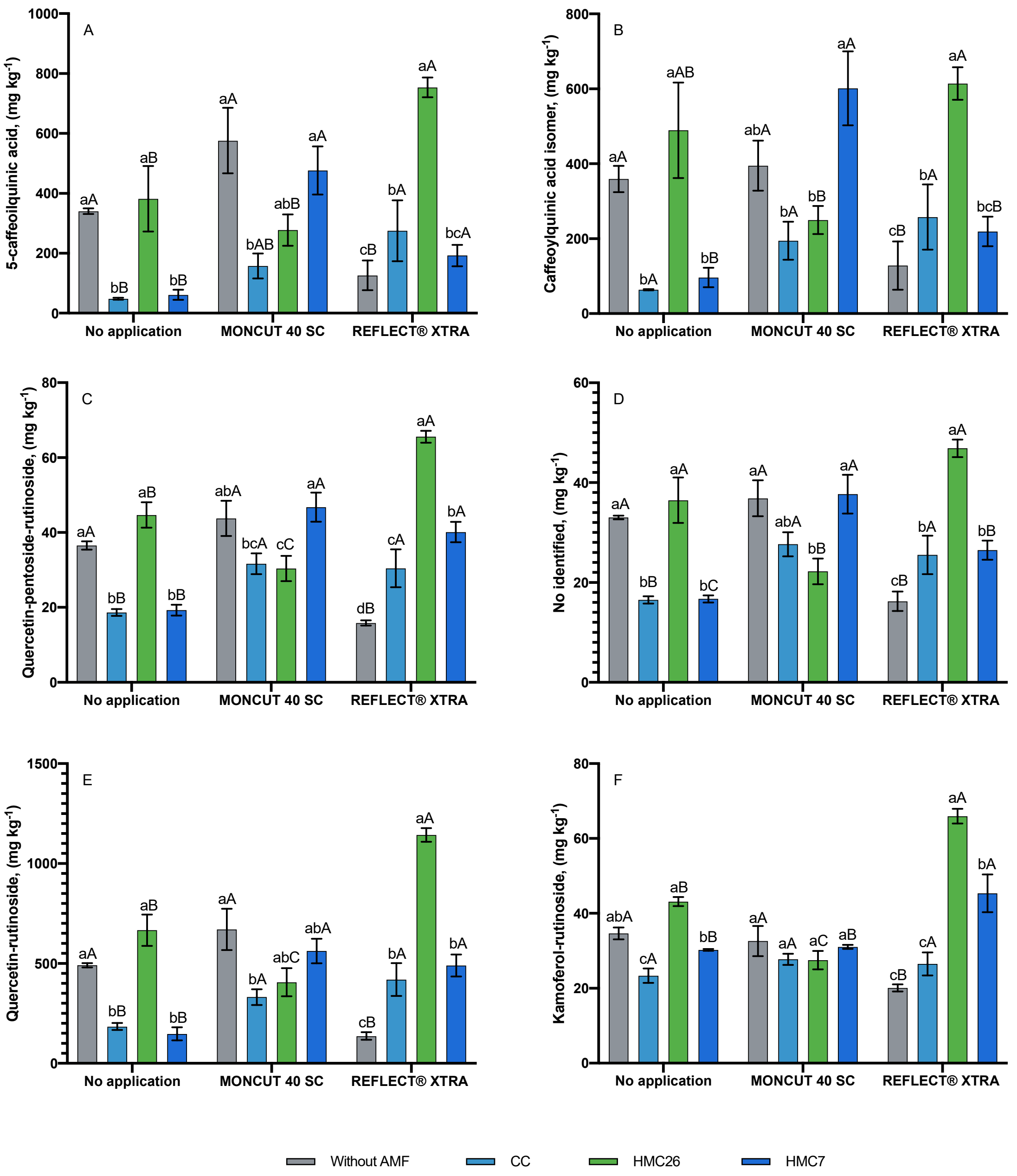

Figure S1. Phenolic compounds concentrations by HPLC-DAD in *Solanum tuberosum* leaves, genotype CB2011-104 under inoculation of arbuscular mycorrhizal fungi and fungicide treatments. Means followed by the same lowercase letter compare inoculation for the same fungicide and uppercase letters compare fungicides within the same inoculation condition (Tukey 5%). (A) 5-caffeoylquinic acid (peak 1), (B) caffeoylquinic acid isomer (peak 2), (C) quercetin-pentoside-rutinoside (peak 5), (D) no identified (peak 6), (E) quercetin-rutinoside (peak 7), (F) kampferol-rutinoside (peak 8).

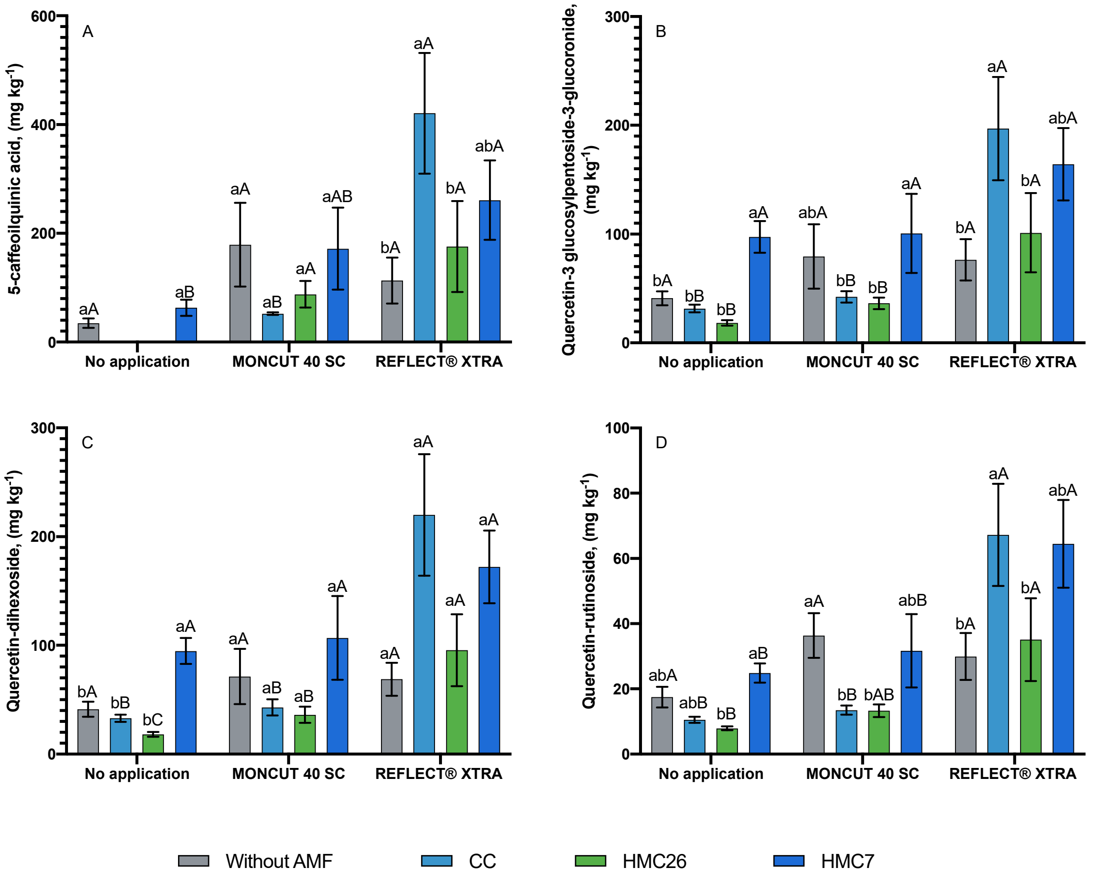

Figure S2. Phenolic compounds concentrations by HPLC-DAD in *Solanum tuberosum* leaves, genotype CB2011-509 under inoculation of arbuscular mycorrhizal fungi and fungicide treatments. Means followed by the same lowercase letter compare inoculation for the same fungicide and uppercase letters compare fungicides within the same inoculation condition (Tukey 5%). (A) 5-caffeoylquinic acid (peak 1), (B) quercetin-3 glucosylrutinoside (peak 3), (C) quercetin-dihexoside (peak 4), (D) quercetin-rutinoside (peak 7).

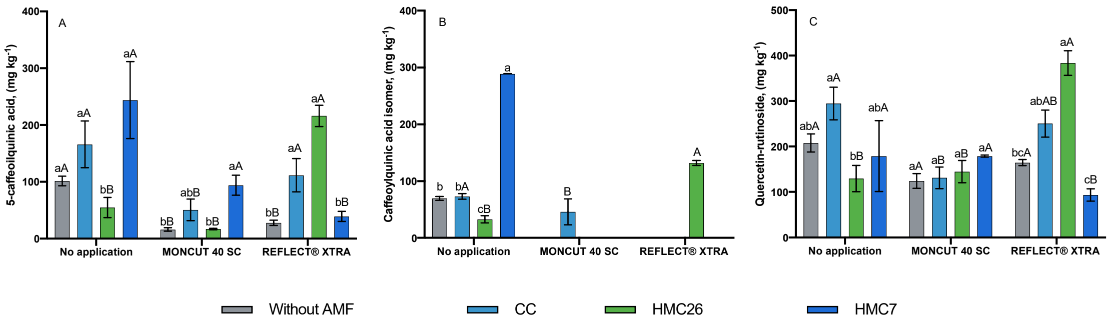

Figure S3. Phenolic compounds concentrations by HPLC-DAD in *Solanum tuberosum* leaves, genotype VR808 under inoculation of arbuscular mycorrhizal fungi and fungicide treatments. Means followed by the same lowercase letter compare inoculation for the same fungicide and uppercase letters compare fungicides within the same inoculation condition (Tukey 5%). (A) 5-caffeoylquinic acid (peak 1,) (B) caffeoylquinic acid isomer (peak 2), (C) quercetin-rutinoside (peak 7).
